# Comprehensive registry of esophageal cancer in Japan, 2014

**DOI:** 10.1007/s10388-021-00879-1

**Published:** 2021-09-22

**Authors:** Masayuki Watanabe, Yasushi Toh, Ryu Ishihara, Koji Kono, Hisahiro Matsubara, Kentaro Murakami, Kei Muro, Hodaka Numasaki, Tsuneo Oyama, Soji Ozawa, Hiroshi Saeki, Koji Tanaka, Takahiro Tsushima, Masaki Ueno, Takashi Uno, Toshiyuki Yoshio, Shiyori Usune, Arata Takahashi, Hiroaki Miyata, The Registration Committee for Esophageal Cancer of the Japan Esophageal Society

**Affiliations:** 1 Department of Gastroenterological Surgery, Cancer Institute Hospital of Japanese Foundation for Cancer Research, 3-8-31 Ariake, Koto-ku, Tokyo, 135-8550 Japan; 2 Department of Gastroenterological Surgery, National Hospital Organization Kyushu Cancer Center, 3-1-1 Notame, Minami-ku, Fukuoka, 811-1395 Japan; 3 Department of Gastrointestinal Oncology, Osaka International Cancer Institute, 3-1-69 Otemae, Chuo-ku, Osaka, 541-8567 Japan; 4 Department of Gastrointestinal Tract Surgery, Fukushima Medical University School of Medicine, 1 Hikarigaoka, Fukushima, 960-1295 Japan; 5 Department of Frontier Surgery, Graduate School of Medicine, Chiba University, 1-8-1 Inohana, Chuo-ku, Chiba, 260-8670 Japan; 6 Department of Clinical Oncology, Aichi Cancer Center Hospital, 1-1 Kanokoden, Chikusa-ku, Nagoya, 464-8681 Japan; 7 Department of Medical Physics and Engineering, Graduate School of Medicine, Osaka University, 2-2 Yamadaoka, Suita, 565-0871 Japan; 8 Department of Endoscopy, Saku Central Hospital Advanced Care Center, 3400-28 Nakagomi, Saku, 385-0051 Japan; 9 Department of Gastroenterological Surgery, Tokai University School of Medicine, 143 Shimokasuya, Isehara, 259-1193 Japan; 10 Department of General Surgical Science, Graduate School of Medicine, Gunma University, 3-39-22 Showa-machi, Maebashi, 371-8511 Japan; 11 Department Gastroenterological Surgery, Graduate School of Medicine, Osaka University, 2-2 Yamadaoka, Suita, 565-0871 Japan; 12 Division of Gastroenterological Oncology, Shizuoka Cancer Center, 1007 Shimonagakubo, Nagaizumi-cho, Sunto-gun, Shizuoka, 411-8777 Japan; 13 Department of Gastroenterological Surgery, Toranomon Hospital, 2-2-2 Toranomon, Minato-ku, Tokyo, 105-8470 Japan; 14 Department of Diagnostic Radiology and Radiation Oncology, Graduate School of Medicine, Chiba University, 1-8-1 Inohana, Chuo-ku, Chiba, 260-8670 Japan; 15 Department of Upper Gastrointestinal Medicine, Cancer Institute Hospital of Japanese Foundation for Cancer Research, 3-8-31 Ariake, Koto-ku, Tokyo, 135-8550 Japan; 16 Department of Healthcare Quality Assessment, Graduate School of Medicine, The University of Tokyo, 7-3-1 Hongo, Bunkyo-ku, Tokyo, 113-8655 Japan

**Keywords:** Esophageal cancer, Esophagectomy, Radiotherapy, Chemotherapy, Endoscopic resection, Chemoradiotherapy

## Abstract

**Background:**

The registration committee for esophageal cancer in the Japan Esophageal Society (JES) has collected the patients' characteristics, treatment, and outcomes annually.

**Methods:**

We analyzed the data of patients who had visited the participating hospitals in 2014. We collected the data with a web-based data collection system using the National Clinical Database. We used the Japanese Classification of Esophageal Cancer 10th edition by JES and the TNM classification 7th edition by the Union of International Cancer Control (UICC) for cancer staging.

**Results:**

A total of 9026 cases were registered from 344 institutions in Japan. Squamous cell carcinoma and adenocarcinoma accounted for 87.9% and 7.1%, respectively. The 5-year survival rates of patients treated using endoscopic resection, concurrent chemoradiotherapy, radiotherapy alone, and esophagectomy were 87.1%, 33.7%, 25.3%, and 59.3%, respectively. Esophagectomy was performed in 5204 cases. Concerning the approach used for esophagectomy, 48.1% of the cases were treated thoracoscopically. The operative mortality (within 30 days after surgery) was 0.75%, and the hospital mortality was 2.0%. The survival curves showed an excellent discriminatory ability both in the clinical and pathologic stages by the JES system. The survival of pStage IV was better than IIIC in the UICC system, because pStage IV included the patients with supraclavicular lymph-node metastasis (M1 LYM).

**Conclusion:**

We hope that this report contributes to improving all aspects of diagnosing and treating esophageal cancer in Japan.

## Preface 2014

We sincerely appreciate the outstanding contributions of many physicians in the registry of esophageal cancer cases. The Comprehensive Registry of Esophageal Cancer in Japan, 2014 was published here. Since 2019, the data collection method was changed from an electronic submission to a web-based data collection using the National Clinical Database (NCD). Personal information was replaced with individual management code inside each institute, and the NCD collected only anonymized information. The registry complies with the Act for the Protection of Personal Information.

We briefly summarized the Comprehensive Registry of Esophageal Cancer in Japan, 2014. According to the subject year, the Japanese Classification of Esophageal Cancer 10th by the Japan Esophageal Society (JES) [1] and the Union of International Cancer Control (UICC) TNM Classification 7th [2] were used for cancer staging. A total of 9026 cases were registered from 344 institutions in Japan. Tumor locations were cervical in 4.8%, upper thoracic in 12.9%, middle thoracic in 46.5%, lower thoracic in 27.2%, and esophagogastric junction in 7.8%. Superficial carcinomas (Tis, T1a, T1b) were 37.2%. As for the histologic type of biopsy specimens, squamous cell carcinoma and adenocarcinoma accounted for 87.9% and 7.1%, respectively. Regarding clinical results, the 5-year survival rates of patients treated using endoscopic resection, concurrent chemoradiotherapy, radiotherapy alone, and esophagectomy were 87.1%, 33.7%, 25.3%, and 59.3%, respectively. The endoscopic submucosal dissection accounted for 92.6% of endoscopic resection. Esophagectomy was performed in 5204 cases. Concerning the approach used for esophagectomy, 48.1% of the cases were treated thoracoscopically. The operative mortality (within 30 days after surgery) was 0.75%, and the hospital mortality was 2.0%. The Kaplan–Meier survival curves diverged according to the N-grade both in the JES and the UICC classifications. The survival curves showed an excellent discriminatory ability both in the clinical and pathologic stages by the JES system. In contrast, in the UICC system, the survival of cStage IIB was better than those of IB and IIA, while the survival curves were almost identical between cStage IIIc and IV. Also, the survival curve of pStage IIB was better than that of IIA, and the survival of pStage IV was better than that of IIIC. pStage IV in the UICC system included the patients with supraclavicular lymph-node metastasis (M1 LYM), which is probably the reason for the better prognosis of pStage IV than pStage IIIC.

We hope that this Comprehensive Registry of Esophageal Cancer in Japan 2014 will help to improve all aspects of the diagnosis and treatment of esophageal cancer in Japan.

## Contents

### I. Clinical factors of esophageal cancer patients treated in 2014

#### 1. Institution-registered cases in 2014

#### 2. Patient background

#### Table 1 Age and gender

**Table 1:** Age and gender

Age	Male	Female	Cases (%)
≤ 29	20	4	24 (0.3)
30–39	22	7	29 (0.3)
40–49	179	74	253 (2.8)
50–59	995	230	1225 (13.6)
60–69	2908	482	3390 (37.6)
70–79	2788	432	3220 (35.7)
80–89	685	148	833 (9.2)
90 ≤	34	18	52 (0.6)
Total	7631	1395	9026

#### Table 2 Performed treatment

**Table 2:** Performed treatment

Treatments	Cases (%)
Surgery	5355 (59.3)
Esophagectomy	5204 (57.7)
Palliative surgery	151 (1.7)
Chemotherapy and/or radiotherapy	4835 (53.6)
Endoscopic treatment	1529 (16.9)

#### Table 3 Tumor location

**Table 3:** Tumor location

Location of tumor	Endoscopic treatment	Surgery		Chemotherapy and/or	Total (%)
	(%)	Esophagectomy (%)	Palliative surgery (%)	radiotherapy (%)	
Cervical	43 (2.8)	185 (3.6)	6 (4.0)	305 (6.3)	436 (4.8)
Upper thoracic	164 (10.7)	598 (11.5)	36 (23.8)	738 (15.3)	1160 (12.9)
Middle thoracic	838 (54.7)	2386 (45.8)	66 (43.7)	2180 (45.1)	4200 (46.5)
Lower thoracic	378 (24.7)	1528 (29.4)	35 (23.2)	1296 (26.8)	2451 (27.2)
EG	68 (4.4)	378 (7.3)	7 (4.6)	214 (4.4)	531 (5.9)
E = G	24 (1.6)	64 (1.2)		30 (0.6)	94 (1.0)
GE	7 (0.5)	62 (1.2)		40 (0.8)	85 (0.9)
Unknown	7 (0.5)	3 (0.1)	1 (0.7)	32 (0.7)	69 (0.8)
Total	1529	5204	151	4835	9026

*E* esophageal, *G* gastric

#### Table 4 Histologic types of biopsy specimens

**Table 4:** Histologic type of biopsy specimens

Histologic types	Endoscopic treatment	Surgery		Chemotherapy and/or	Total (%)
	(%)	Esophagectomy (%)	Palliative surgery (%)	radiotherapy (%)	
Squamous cell carcinoma	1314 (85.9)	4567 (87.8)	143 (94.7)	4450 (92.0)	7938 (87.9)
Squamous cell carcinoma	993 (64.2)	2484 (47.7)	93 (61.6)	2601 (53.8)	4819 (53.4)
Well differentiated	104 (6.8)	427 (8.2)	12 (7.9)	320 (6.6)	640 (7.1)
Moderately differentiated	172 (11.2)	1234 (23.7)	29 (19.2)	1098 (22.7)	1807 (20.0)
Poorly differentiated	45 (2.9)	422 (8.1)	9 (6.0)	431 (8.9)	672 (7.4)
Adenocarcinoma	41 (2.7)	372 (7.1)	3 (2.0)	199 (4.1)	492 (5.5)
Barrett's carcinoma	42 (2.7)	96 (1.8)	1 (0.7)	25 (0.5)	144 (1.6)
Adenosquamous carcinoma	1 (0.1)	10 (0.2)		7 (0.1)	18 (0.2)
Mucoepidermoid carcinoma		2 (0.0)		1 (0.0)	3 (0.0)
Basaloid carcinoma	4 (0.3)	32 (0.6)		19 (0.4)	41 (0.5)
Neuroendocrine tumor				1 (0.0)	1 (0.0)
Neuroendocrine carcinoma	1 (0.1)	16 (0.3)		34 (0.7)	41 (0.5)
Undifferentiated carcinoma	1 (0.1)	4 (0.1)		2 (0.0)	5 (0.3)
Malignant melanoma		18 (0.3)		9 (0.2)	24 (0.3)
Carcinosarcoma	1 (0.1)	22 (0.4)		12 (0.2)	28 (0.3)
GIST		7 (0.1)		2 (0.0)	8 (0.1)
Adenoid cystic carcinoma		1 (0.0)			1 (0.0)
Nonepithelial tumors	2 (0.1)	3 (0.1)		3 (0.1)	6 (0.1)
Other epithelial tumors	36 (2.4)	8 (0.2)		9 (0.2)	58 (0.6)
Other tumors	26 (1.7)	15 (0.3)		5 (0.1)	47 (0.5)
Unknown	60 (3.9)	31 (0.6)	4 (2.6)	57 (2.1)	171 (1.9)
Total	1529	5204	151	4835	9026

#### Table 5 Depth of tumor invasion, cT (UICC TNM 7th)

**Table 5:** Depth of tumor invasion, cT (UICC TNM 7th)

Clinical T	Endoscopic treatment	Surgery		Chemotherapy and/or	Total (%)
	(%)	Esophagectomy (%)	Palliative surgery (%)	radiotherapy (%)	
cTX	28 (1.8)	17 (0.3)	4 (2.6)	57 (1.2)	144 (1.6)
cT0	17 (1.1)	7 (0.1)		3 (0.1)	30 (0.3)
cT1a	1173 (76.7)	240 (4.6)		112 (2.3)	1469 (16.3)
cT1b	205 (13.4)	1409 (27.1)	2 (1.3)	644 (13.3)	1858 (20.6)
cT2	9 (0.6)	867 (16.7)	5 (3.3)	667 (13.8)	1086 (12.0)
cT3	46 (3.0)	2310 (44.4)	62 (41.1)	2367 (49.0)	3250 (36.0)
cT4a	10 (0.7)	164 (3.2)	13 (8.6)	317 (6.6)	404 (4.5)
cT4b	41 (2.7)	190 (3.7)	65 (43.0)	668 (13.8)	785 (8.7)
Total	1529	5204	151	4835	9026

#### Table 6 Lymph-node metastasis, cN (UICC TNM 7th)

**Table 6:** Lymph-node metastasis, cN (UICC TNM 7th)

Clinical N	Endoscopic treatment	Surgery		Chemotherapy and/or	Total (%)
	(%)	Esophagectomy (%)	Palliative surgery (%)	radiotherapy (%)	
cN0	1426 (93.3)	2390 (45.9)	20 (13.2)	1310 (27.1)	4399 (48.7)
cN1	50 (3.3)	1825 (35.1)	60 (39.7)	1914 (39.6)	2627 (29.1)
cN2	33 (2.2)	867 (16.7)	56 (37.1)	1257 (26.0)	1567 (17.4)
cN3	20 (1.3)	122 (2.3)	15 (9.9)	354 (7.3)	433 (4.8)
Total	1529	5204	151	4835	9026

#### Table 7 Distant metastasis, cM (UICC TNM 7th)

**Table 7:** Distant metastasis, cM (UICC TNM 7th)

Clinical M	Endoscopic treatment	Surgery		Chemotherapy and/or	Total (%)
	(%)	Esophagectomy (%)	Palliative surgery (%)	radiotherapy (%)	
cM0	1494 (97.7)	5036 (96.8)	108 (71.5)	4210 (85.2)	8148 (90.3)
cM1	35 (2.3)	168 (3.2)	43 (28.5)	715 (14.8)	878 (9.7)
Total	1529	5204	151	4835	9026

#### Table 8 Clinical stage (UICC TNM 7th)

**Table 8:** Clinical Stage (UICC TNM 7th)

Clinical stage	Endoscopic treatment	Surgery		Chemotherapy and/or	Total (%)
	(%)	Esophagectomy (%)	Palliative surgery (%)	radiotherapy (%)	
Stage IA	1363 (89.1)	1307 (25.1)	2 (1.3)	471 (9.7)	2899 (32.1)
Stage IB	5 (0.3)	458 (8.8)	2 (1.3)	282 (5.8)	558 (6.2)
Stage IIA	10 (0.7)	531 (10.2)	6 (4.0)	400 (8.3)	649 (7.2)
Stage IIB	15 (1.0)	577 (11.1)	1 (0.7)	449 (9.3)	680 (7.5)
Stage IIIA	14 (0.9)	1195 (23.0)	21 (13.9)	1078 (22.3)	1499 (16.6)
Stage IIIB	8 (0.5)	560 (10.8)	16 (10.6)	567 (11.7)	733 (8.1)
Stage IIIC	35 (2.3)	385 (7.4)	57 (37.7)	839 (17.4)	997 (11.0)
Stage IV	35 (2.3)	168 (3.2)	43 (28.5)	715 (14.8)	878 (9.7)
Unknown	44 (2.9)	23 (0.4)	3 (2.0)	34 (0.7)	133 (1.5)
Total	1529	5204	151	4835	9026

### II. Results of endoscopically treated patients in 2014

#### Table 9 Details of endoscopic treatment for curative intent

**Table 9:** Details of endoscopic treatment for curative intent

Treatment details	Cases (%)
EMR	104 (7.1)
EMR + YAG laser	1 (0.1)
EMR + MCT/RFA	
ESD	1265 (86.0)
ESD + EMR	80 (5.4)
ESD + PDT	
ESD + YAG laser	2 (0.1)
PDT	3 (0.2)
YAG laser	16 (1.1)
Total	1471

*EMR* endoscopic mucosal resection, *PDT* photodynamic therapy, *YAG* yttrium aluminum garnet, *MCT* microwave coagulation therapy, *ESD* endoscopic submucosal dissection

#### Table 10 Complications of EMR/ESD

**Table 10:** Complications of EMR/ESD

Complications of EMR/ESD	Cases (%)
None	1384 (95.8)
Perforation	12 (0.8)
Bleeding	3 (0.2)
Mediastinitis	5 (0.3)
Stenosis	41 (2.8)
Others	
Unknown	
Total	1445

#### Table 11 Pathologic depth of tumor invasion of EMR/ESD specimens

**Table 11:** Pathologic depth of tumor invasion of MER/ESD specimens

Pathological depth of tumor invasion (pT)	Cases (%)
pTX	17 (1.2)
pT0	68 (0.5)
pT1a	1127 (82.8)
pT1b	238 (15.0)
pT2	
pT3	2 (0.1)
Total	1452

#### Figure 1 Survival of patients treated with EMR/ESD

**Fig. 1:**
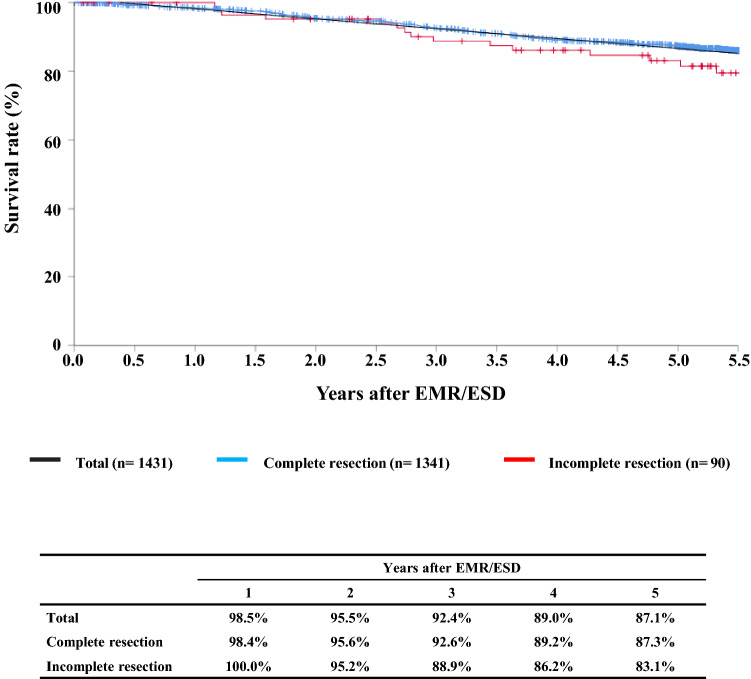
Survival of patients treated with EMR/ESD

#### Figure 2 Survival of patients treated with EM/ESD according to the pathological depth of tumor invasion, pT (JES 10th)

**Fig. 2:**
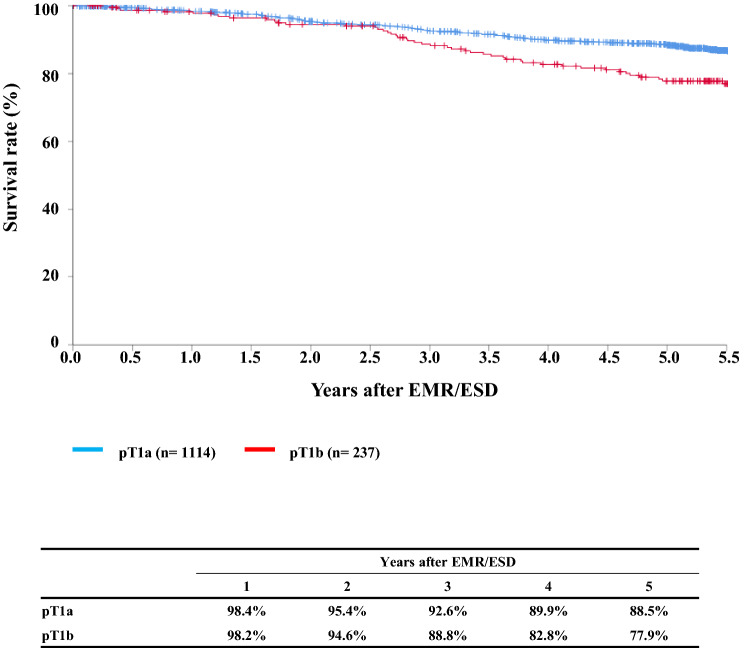
Survival of patients treated with EM/ESD according to the pathological depth of tumor invasion, pT (JES 10th)

#### Figure 3 Survival of patients treated with EMR/ESD according to the lymphatic and venous invasion

**Fig. 3:**
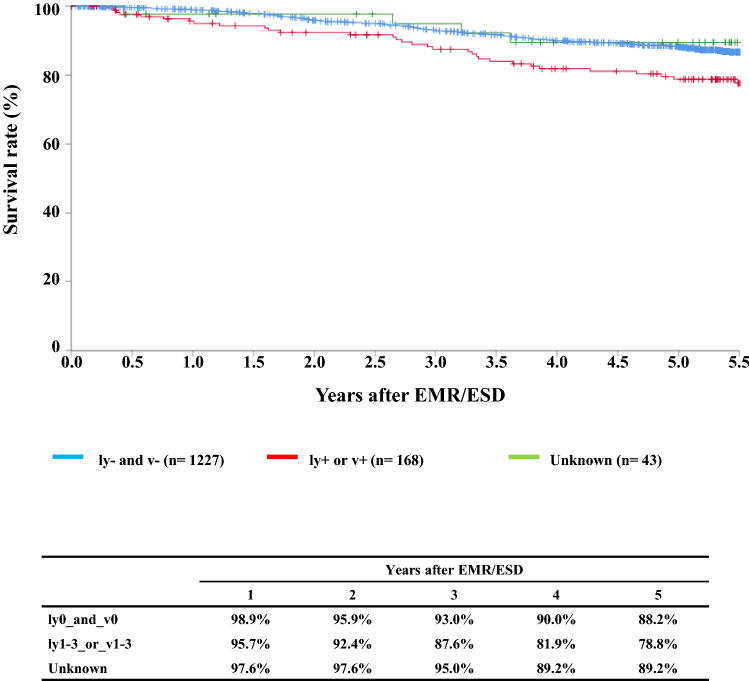
Survival of patients treated with EMR/ESD according to the lymphatic and venous invasion

### III. Results in patients treated with chemotherapy and/or radiotherapy in 2014

#### Table 12 Dose of irradiation (non-surgically treated cases)

**Table 12:** Dose of irradiation (non-surgically treated cases)

Dose of irradiation	Definitive		Palliative (%)	Recurrence (%)	Others (%)	Total (%)
(Gy)	Radiation alone (%)	With chemotherapy (%)				
-29	2 (1.2)	16 (1.7)	26 (8.4)	2 (6.3)	3 (37.5)	49 (3.3)
30–39	3 (1.8)	17 (1.8)	53 (17.1)	5 (15.6)		78 (5.6)
40–49	5 (3.0)	34 (3.5)	56 (18.1)	4 (12.5)	2 (25.0)	101 (6.8)
50–59	26 (15.8)	246 (25.5)	77 (24.8)	8 (25.0)	1 (12.5)	359 (24.2)
60–69	124 (75.2)	620 (64.4)	90 (29.0)	11 (34.4)	2 (25.0)	849 (57.3)
70-	4 (2.4)	28 (2.9)	5 (1.6)	2 (.3)		39 (2.6)
Unknown	1 (0.6)	2 (0.2)	3 (1.0)			6 (0.4)
Total	165	963	310	32	8	1481
Median (min–max)	60.0 (10.0–70.0)	60.0 (2.0–92.0)	50.0 (2.0–90.0)	50.4 (8.0–70.0)	60.0 (50.0–63.4)	60.0 (2.0–92.0)

#### Table 13 Dose of irradiation (surgically treated cases)

**Table 13:** Dose of irradiation (surgically treated cases)

Dose of	Preoperative	Postoperative
irradiation (Gy)	irradiation (%)	irradiation (%)
-29	12 (3.7)	
30–39	55 (16.9)	3 (5.0)
40–49	199 (61.0)	9 (15.0)
50–59	40 (12.3)	20 (33.3)
60–69	16 (4.9)	24 (40.0)
70-	1 (0.3)	3 (5.0)
Unknown	3 (0.9)	1 (1.7)
Total	326	60
Median (min—max)	40.0 (1.8- 70.0)	54.0 (30.0 – 97.5)

#### Figure 4 Survival of patients treated with chemotherapy and/or radiotherapy

**Fig. 4:**
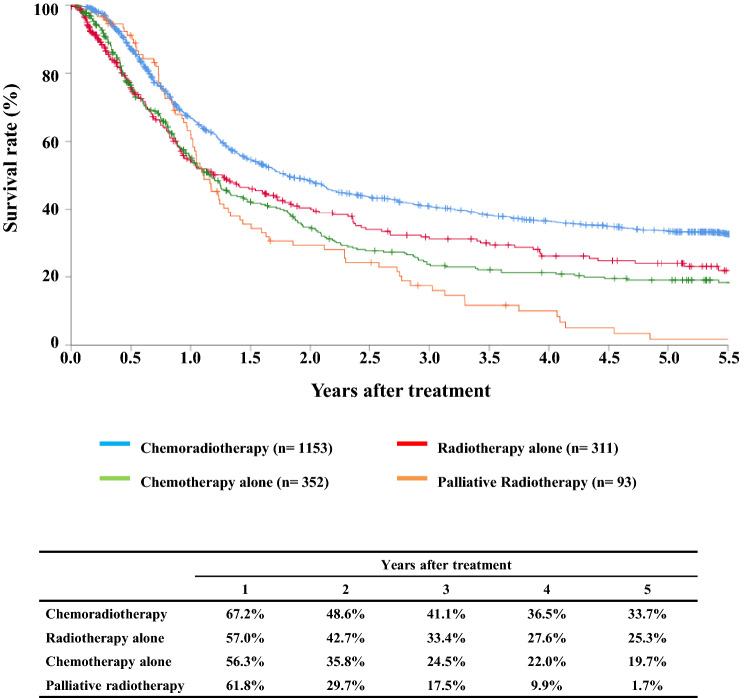
Survival of patients treated with chemotherapy and/or radiotherapy

#### Figure 5 Survival of patients treated with definitive chemoradiotherapy according to the clinical stage (UICC TNM 7th)

**Fig. 5:**
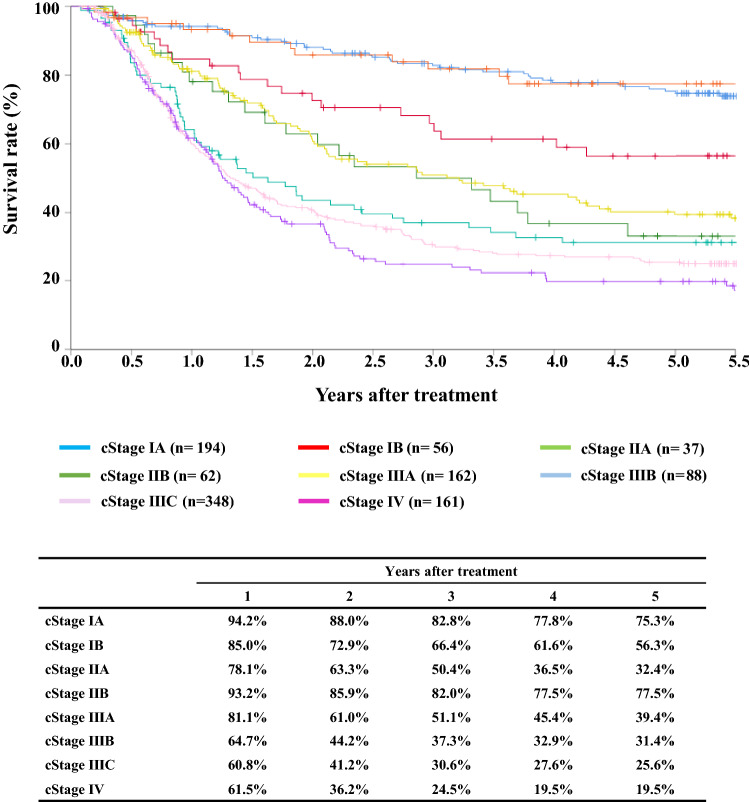
Survival of patients treated with definitive chemoradiotherapy according to the clinical stage (UICC TNM 7th)

#### Figure 6 Survival of patients underwent radiotherapy alone according to the clinical stage ( UICC TNM 7th)

**Fig. 6:**
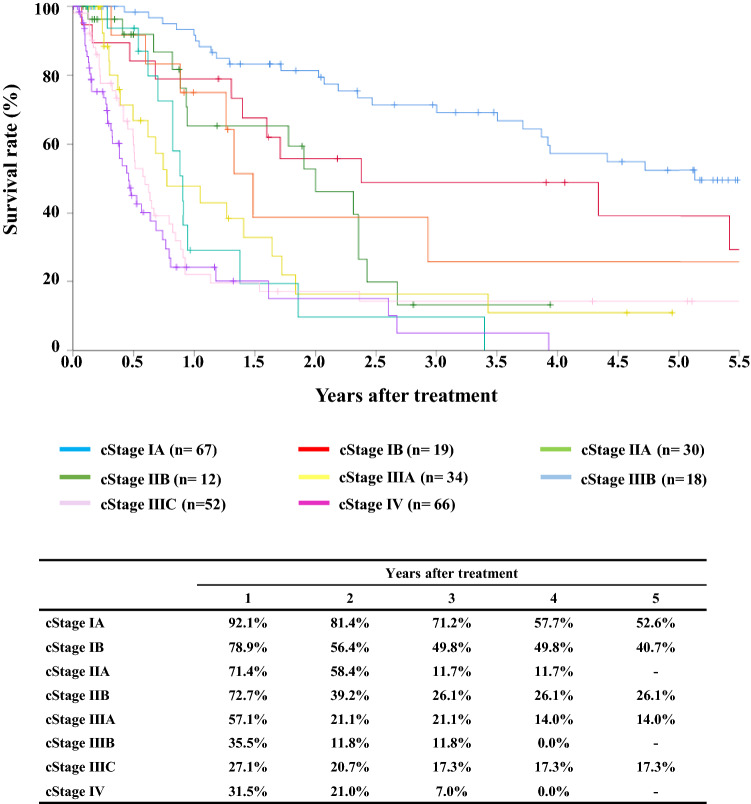
Survival of patients who underwent radiotherapy alone according to the clinical stage (UICC TNM 7th)

### IV. Results in patients who underwent esophagectomy in 2014

#### Table 14 Treatment modalities of esophagectomy

**Table 14:** Treatment modalities of esophagectomy

Treatment modalities	Cases (%)
Esophagectomy alone	2307 (44.3)
Esophagectomy + postoperative chemotherapy	387 (7.4)
Esophagectomy + postoperative chemoradiotherapy	109 (2.1)
Esophagectomy + postoperative radiotherapy	39 (0.7)
Preoperative chemotherapy + Esophagectomy	1784 (34.3)
Preoperative chemoradiotherapy + Esophagectomy	335 (6.4)
Definitive radiotherapy + Esophagectomy	6 (0.1)
Definitive chemoradiotherapy + Esophagectomy	124 (2.4)
Others	113 (2.2)
Total	5204

#### Table 15 Tumor location

**Table 15:** Tumor location

Locations	Cases (%)
Cervical	209 (3.8)
Upper thoracic	655 (12.0)
Middle thoracic	2448 (44.9)
Lower thoracic	1570 (28.8)
EG	380 (7.0)
E = G	98 (1.8)
GE	80 (1.5)
Unknown	11 (0.2)
Total	5451

#### Table 16 Approaches to tumor resection

**Table 16:** Approaches to tumor resection

Approaches	Cases (%)
Cervical	176 (3.4)
Right thoracic	4492 (86.3)
Left thoracic	54 (1.0)
Left thoracoabdominal	82 (1.6)
Abdominal	187 (3.6)
Transhiatal lower esophagectomy	133 (2.6)
Transhiatal thoracic esophagectomy	64 (1.2)
Sternotomy	6 (0.1)
Others	7 (0.1)
Unknown	3 (0.1)
Total	5204

Thoracic includes thoracotomy and thoracoscopic. Abdominal includes laparotomy and laparoscopic

#### Table 17 Video-assisted surgery

**Table 17:** Video-assisted surgery

Video-assisted surgery	Cases (%)
None	2330 (44.6)
Thoracoscopy	1206 (23.2)
Thoracoscopy + laparoscopy	1281 (24.6)
Thoracoscopy + laparoscopy + mediastinoscopy	9 (0.2)
Thoracoscopy + laparoscopy + other	
Thoracoscopy + mediastinoscopy	1 (0.0)
Thoracoscopy + other	4 (0.1)
Laparoscopy	265 (5.1)
Laparoscopy + mediastinoscopy	41 (0.8)
Laparoscopy + mediastinoscopy + other	1 (0.0)
Mediastinoscopy	49 (0.9)
Laparoscopy + other	1 (0.0)
Others	15 (0.3)
Unknown	1 (0.0)
Total	5204

#### Table 18 Fields of lymph-node dissection according to the location of the tumor

**Table 18:** Fields of lymph-node dissection according to the location of tumor

Field of lymphadenectomy	Cervical	Upper thoracic	Middle thoracic	Lower thoracic	Abdominal	E = G	GE	Unknown	Total
None	8 (4.2)	15 (2.5)	46 (1.9)	26 (1.7)	4 (1.1)	1 (1.1)	4 (6.0)		104 (2.0)
C	47 (24.5)	11 (1.8)	33 (1.4)	14 (0.9)					105 (2.0)
C + UM	21 (10.9)	1 (0.2)	2 (0.1)		1 (0.3)				25 (0.5)
C + UM + MLM	4 (2.1)	21 (3.4)	50 (2.1)	12 (0.8)	1 (0.3)				88 (1.7)
C + UM + MLM + A	83 (43.2)	394 (64.6)	1205 (64.6)	577 (37.7)	43 (12.0)	6 (6.8)	6 (9.0)	1 (50.0)	2315 (44.5)
C + UM + A	6 (3.1)	10 (1.6)	22 (0.9)	10 (0.7)	1 (0.3)				49 (0.9)
C + MLM	1 (0.5)	1 (0.2)							2 (0.0)
C + MLM + A	1 (0.5)	3 (0.5)	15 (0.4)	6 (0.4)	3 (0.8)	1 (1.1)			29 (0.6)
C + A	4 (2.1)	1 (0.2)	1 (0.0)	2 (0.1)					8 (0.2)
UM	2 (1.0)	4 (0.7)	11 (0.5)	2 (0.1)					19 (0.4)
UM + MLM	3 (1.6)	8 (1.3)	40 (1.7)	27 (1.8)	4 (1.1)				82 (1.6)
UM + MLM + A	5 (2.6)	125 (20.5)	847 (35.9)	675 (44.1)	115 (32.2)	24 (27.3)	3 (4.5)	1 (50.0)	1795 (34.5)
UM + A		5 (0.8)	14 (0.6)	9 (0.6)	3 (0.8)	1 (1.1)			32 (0.6)
MLM		2 (0.3)	11 (0.5)	15 (1.0)	3 (0.8)	2 (2.3)	1 (1.5)		34 (0.7)
MLM + A		4 (0.7)	48 (2.0)	130 (8.5)	139 (38.9)	39 (44.3)	33 (49.3)		399 (7.7)
A	1 (0.5)	5 (0.8)	14 (0.6)	24 (1.6)	40 (11.2)	14 (15.9)	20 (29.9)		118 (2.3)
Total	192	610	2359	1529	357	88	67	2	5204

*C* bilateral cervical nodes, *UM* upper mediastinal nodes, *MLM* middle-lower mediastinal nodes, *A* abdominal nodes

#### Table 19 Reconstruction route

**Table 19:** Reconstruction route

Route	Cases (%)
None	47 (0.9)
Subcutaneous	345 (6.6)
Retrosternal	2315 (44.5)
Posterior mediastinal	1920 (36.9)
Intrathoracic	465 (8.9)
Cervical	65 (1.2)
Others	41 (0.8)
Unknown	6 (0.1)
Total	5204

#### Table 20 Organs used for reconstruction

**Table 20:** Organs used for reconstruction

Organs	Cases (%)
None	85 (1.6)
Whole stomach	105 (2.0)
Gastric tube	4425 (84.3)
Jejunum	272 (5.2)
Free jejunum	119 (2.3)
Colon	197 (3.8)
Free colon	10 (0.2)
Others	36 (0.7)
Total organs	5249
Total cases	5119

#### Table 21 Histological classification

**Table 21:** Histological classification

Histological classification	Cases (%)
Squamous cell carcinoma	4324 (83.1)
Squamous cell carcinoma	751 (14.4)
Well differentiated	764 (14.7)
Moderately differentiated	2172 (41.7)
Poorly differentiated	637 (12.2)
Adenocarcinoma	347 (6.7)
Barrett's carcinoma	113 (2.2)
Adenosquamous carcinoma	29 (0.6)
Mucoepidermoid carcinoma	6 (0.1)
Basaloid carcinoma	82 (1.6)
Neuroendocrine tumor	2 (0.0)
Neuroendocrine carcinoma	25 (0.5)
Undifferentiated carcinoma	5 (0.1)
Malignant melanoma	19 (0.4)
Carcinosarcoma	37 (0.7)
GIST	7 (0.1)
Adenoid cystic carcinoma	1 (0.0)
Sarcoma	2 (0.0)
Other carcinomas	8 (0.2)
Other tumors	54 (1.0)
Unknown	143 (2.7)
Total	5204

#### Table 22 Depth of tumor invasion, pT (JES 10th)

**Table 22:** Pathological depth of tumor invasion, pT (JES 10th)

Pathological depth of tumor invasion	Cases (%)
pTx	42 (0.8)
pT0	227 (4.4)
pT1a	645 (12.4)
pT1b	1475 (28.3)
pT2	590 (11.3)
pT3	1962 (37.7)
pT4a	141 (2.7)
pT4b	122 (2.3)
Total	5204

#### Table 23 Pathological grading of lymph-node metastasis, pN (JES 10th)

**Table 23:** Pathological grading of lymph-node metastasis, pN (JES 10th)

Lymph-node metastasis	Cases (%)
pN0	2568 (49.3)
pN1	962 (18.5)
pN2	966 (18.6)
pN3	371 (7.1)
pN4	321 (6.2)
Unknown	16 (0.3)
Total	5204

#### Table 24 Pathological findings of lymph-node metastasis, pN (UICC TNM 7th)

**Table 24:** Pathological grading of lymph-node metastasis, pN (UICC TNM 7th)

Lymph-node metastasis	Cases (%)
pN0	2611 (50.2)
pN1 (1–2)	1397 (26.8)
pN2 (3–6)	787 (15.1)
pN3 (7-)	373 (7.2)
Unknown	36 (0.7)
Total	5204

#### Table 25 Pathological findings of distant organ metastasis, pM (JES 10th)

**Table 25:** Pathological findings of distant organ metastasis, pM (JES 10th)

Distant metastasis (M)	Cases (%)
MX	110 (2.1)
M0	4998 (96.0)
M1	96 (1.8)
Total	5204

#### Table 26 Residual tumor

**Table 26:** Residual tumor

Residual tumor (R)	Cases (%)
RX	95 (1.8)
R0	4663 (89.6)
R1	257 (4.9)
R2	189 (3.6)
Total	5204

#### Figure 7 Survival of patients who underwent esophagectomy

**Fig. 7:**
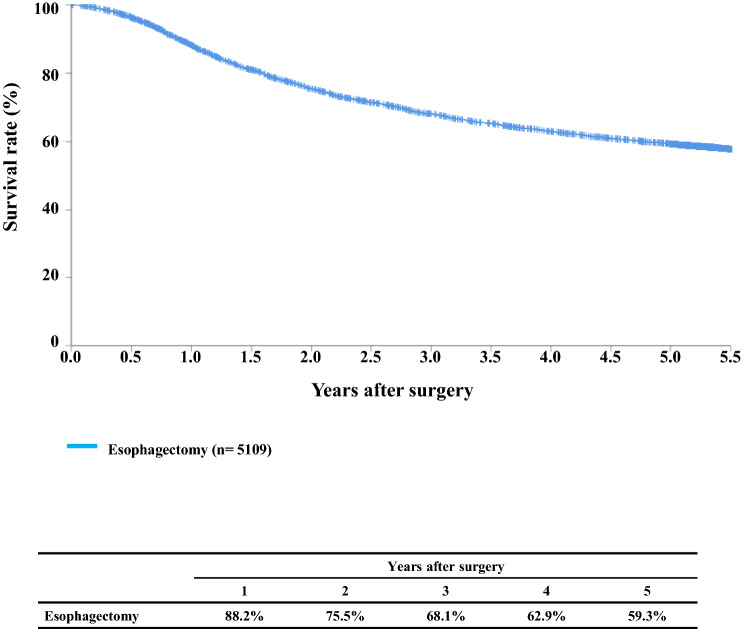
Survival of patients who underwent esophagectomy

#### Figure 8 Survival of patients who underwent esophagectomy according to the clinical stage (JES 10th)

**Fig. 8:**
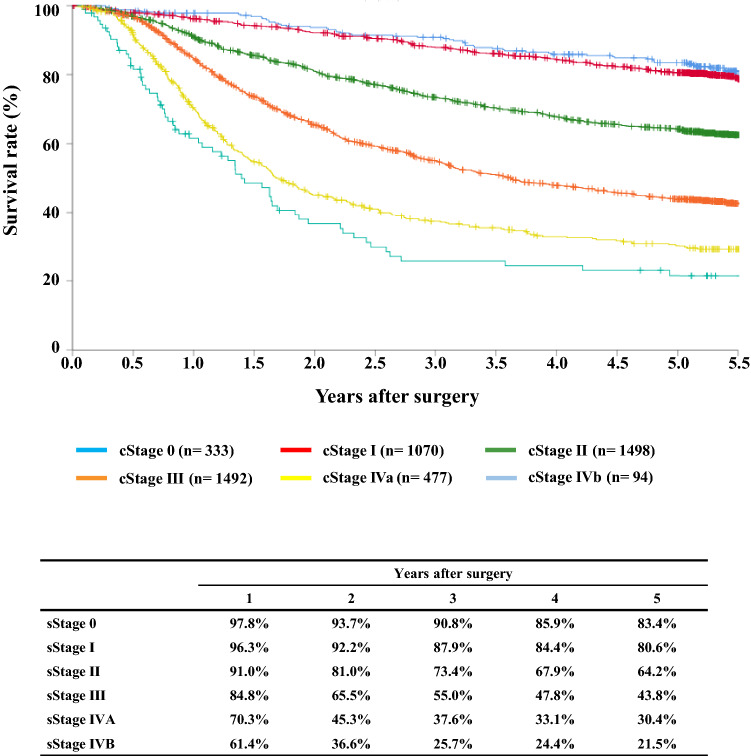
Survival of patients who underwent esophagectomy according to the clinical stage (JES 10th)

#### Figure 9 Survival of patients who underwent esophagectomy according to the clinical stage (UICC TNM 7th)

**Fig. 9:**
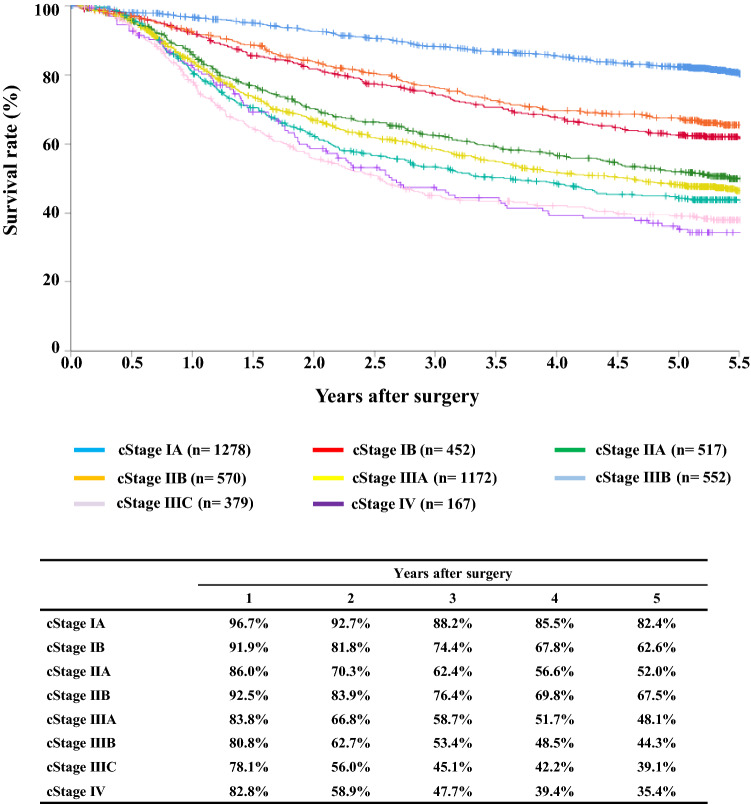
Survival of patients who underwent esophagectomy according to the clinical stage (UICC TNM 7th)

#### Figure 10 Survival of patients who underwent esophagectomy according to the depth of tumor invasion, pT (JES 10th)

**Fig. 10:**
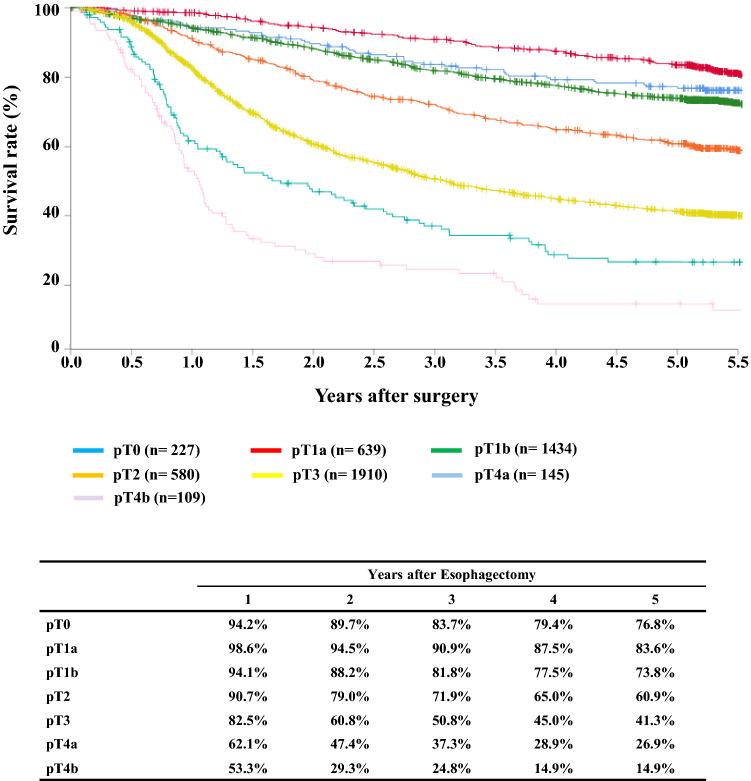
Survival of patients who underwent esophagectomy according to the depth of tumor invasion, pT (JES 10th)

#### Figure 11 Survival of patients who underwent esophagectomy according to lymph node metastasis (JES 10th)

**Fig. 11:**
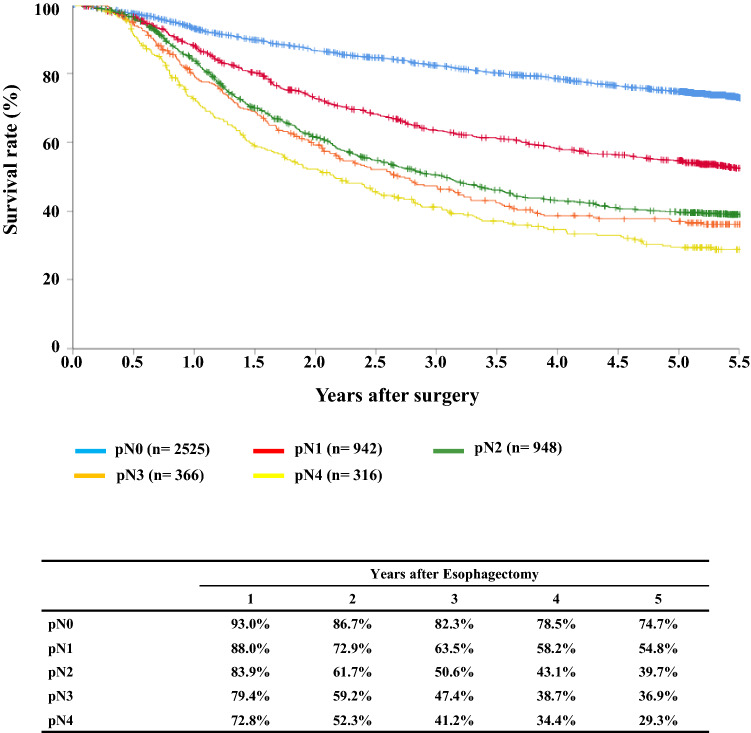
Survival of patients who underwent esophagectomy according to lymph-node metastasis (JES 10th)

#### Figure 12 Survival of patients who underwent esophagectomy according to lymph node metastasis (UICC TNM 7th)

**Fig. 12:**
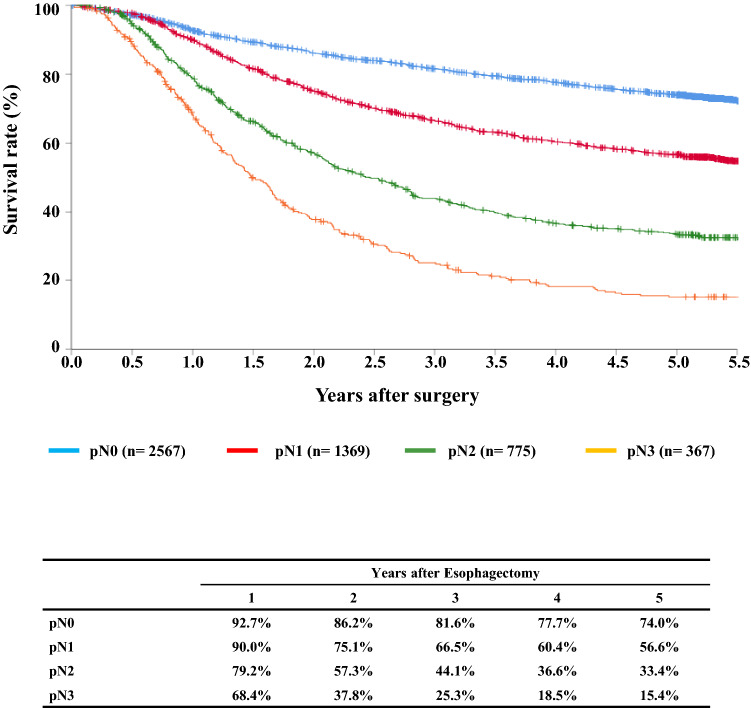
Survival of patients who underwent esophagectomy according to lymph-node metastasis (UICC TNM 7th)

#### Figure 13 Survival of patients who underwent esophagectomy according to the pathological stage (JES 10th)

**Fig. 13:**
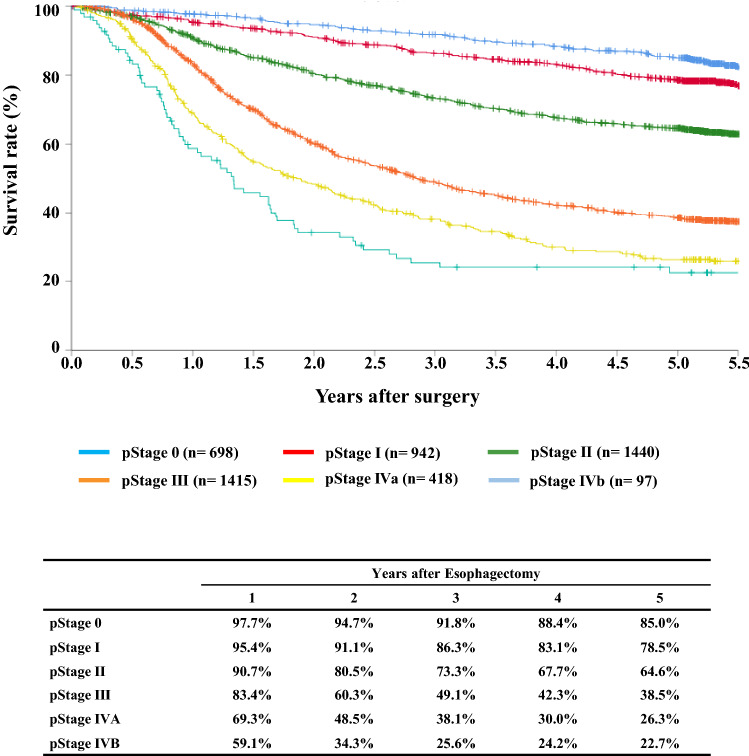
Survival of patients who underwent esophagectomy according to the pathological stage (JES 10th)

#### Figure 14 Survival of patients who underwent esophagectomy according to the pathological stage (UICC TNM 7th)

**Fig. 14:**
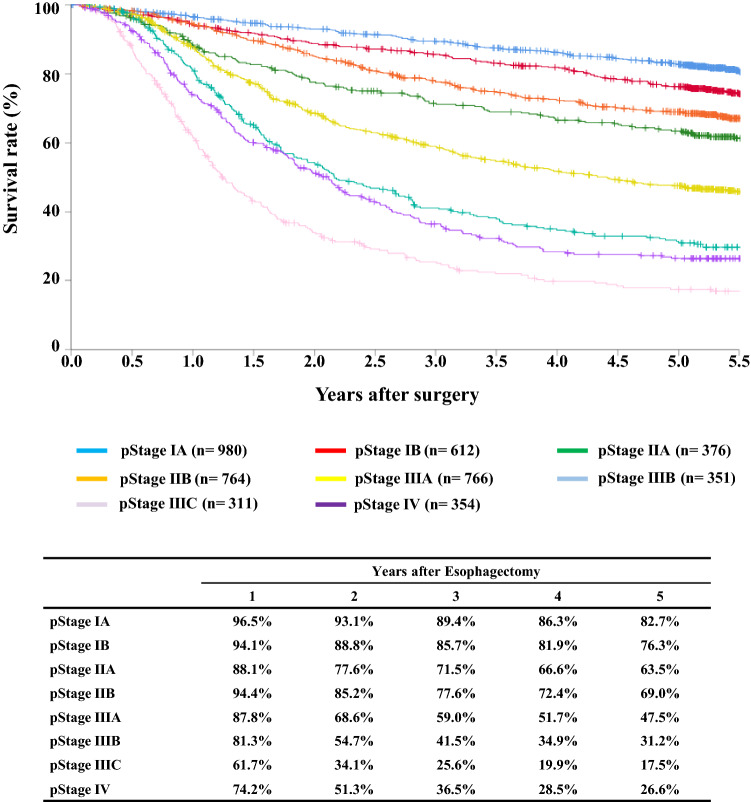
Survival of patients who underwent esophagectomy according to the pathological stage (UICC TNM 7th)

#### Figure 15 Survival of patients who underwent esophagectomy according to the residual tumor (R)

**Fig. 15:**
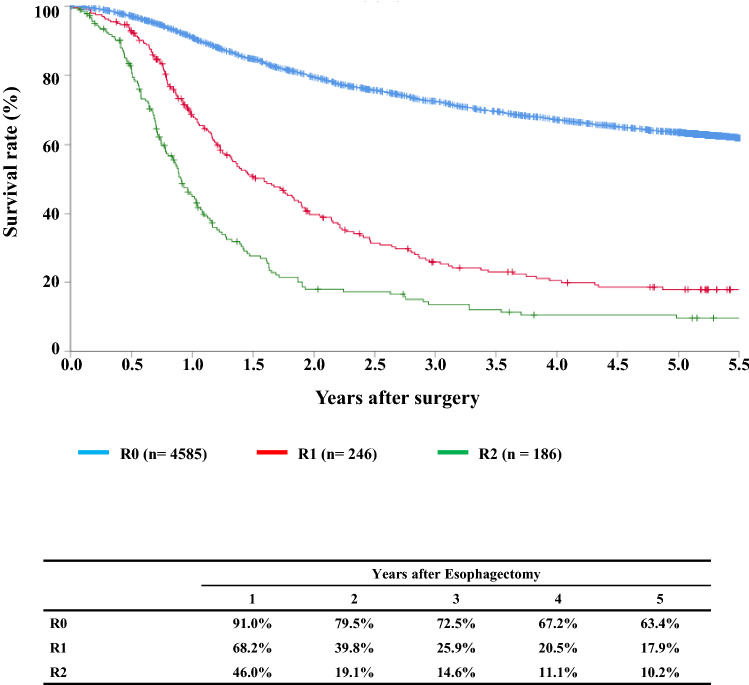
Survival of patients

## I. Clinical features of esophageal cancer patients treated in 2014

Institution-registered cases in 2014.

**Table:** 

Institutions
Ageo Central General Hospital
Aichi Cancer Center
Aichi Medical University Hospital
Aizawa Hospital
Akita University Hospital
Arao Municipal Hospital
Asahi Rousai Hospital
Asahikawa Medical University Hospital
Cancer Institute Hospital of JFCR
Chiba Cancer Center
Chiba University Hospital
Chiba-ken Saiseikai Narashino Hospital
Dokkyo Medical University Hospital
Dokkyo Medical University Saitama Medical Center
Edogawa Hospital
Ehime Prefectural Central Hospital
Eijyu General Hospital
Fuchu Hospital
Fuji City General Hospital
Fujinomiya City General Hospital
Fujioka General Hospital
Fujisaki Hospital
Fujita Health University Hospital
Fukaya Red Cross Hospital
Fukui University Hospital
Fukui-ken Saiseikai Hospital
Fukuoka City Hospital
Fukuoka Shin Mizumaki Hospital
Fukuoka University Chikushi Hospital
Fukuoka University Hospital
Fukushima Medical University Hospital
Fukuyama City Hospital
Fussa Hospital
Gifu Prefectural General Center
Gifu University Hospital
Gunma Prefectural Cancer Center
Gunma Saiseikai Maebashi Hospital
Gunma University Hospital
Hachinohe City Hospital
Hagi City Hospital
Hakodate City Hospital
Hakodate Goryokaku Hospital
Hakodate National Hospital
Hamamatsu University Hospital
Hannan Chuo Hospital
Hasuda Hospital
Heartlife Hospital
Higashiosaka City Medical Center
Hiraka General Hospital
Hiratsuka City Hospital
Hirosaki University Hospital
Hiroshima City Asa Hospital
Hiroshima City Hospital
Hiroshima Prefectural Hospital
Hiroshima Red Cross Hospital & Atomic-Bomb Survivors Hospital
Hiroshima University Hospital
Hitachi General Hospital
Hofu Institute of Gastroenterology
Hokkaido University Hospital
Hospital of the University of Occupational and Environmental Health, Japan
Hyogo Cancer Center
Hyogo Prefectural Amagasaki General Medical Center
Hyogo Prefectural Nishinomiya Hospital
Ibaraki Prefectural Central Hospital
Iizuka Hospital
Ikeda City Hospital
Imari Arita Kyoritsu Hospital
International University of Health and Welfare Atami Hospital
International University of Health and Welfare Hospital
International University of Health and Welfare Mita Hospital
Isehara Kyodo Hospital
Iseikai Hospital
Ishikawa Prefectural Central Hospital
Itami City Hospital
Iwata City Hospital
Iwate Medical University Hospital
Iwate Prefectural Central Hospital
Iwate Prefectural Chubu Hospital
JA Hiroshima General Hospital
JA Kouseiren Enshu Hospital
JA Onomichi General Hospital
Japanese Red Cross Ashikaga Hospital
Japanese Red Cross Fukuoka Hospital
Japanese Red Cross Ishinomaki Hospital
Japanese Red Cross Kitami Hospital
Japanese Red Cross Kyoto Daiichi Hospital
Japanese Red Cross Maebashi Hospital
Japanese Red Cross Medical Center
Japanese Red Cross Musashino Hospital
Japanese Red Cross Nagoya Daiichi Hospital
Japanese Red Cross Nagoya Daini Hospital
Japanese Red Cross Saitama Hospital
Japanese Red Cross Tottori Hospital
Japanese Red Cross Wakayama Medical Center
Japanese Red Cross Yamaguchi Hospital
JCHO Gunma Chuo Hospital
JCHO Kyushu Hospital
JCHO Osaka Hospital
JCHO Saitama Medical Center
Jichi Medical University Hospital
Jichi Medical University Saitama Medical Center
Juntendo University Hospital
Juntendo University Nerima Hospital
Juntendo University Shizuoka Hospital
Juntendo University Urayasu Hospital
Junwakai Memorial Hospital
Kagawa Prefectural Central Hospital
Kagawa Rosai Hospital
Kagawa University Hospital
Kagoshima City Hospital
Kagoshima University Hospital
Kakogawa Central City Hospital
Kanagawa Cancer Center
Kanagawa Prefectural Ashigarakami Hospital
Kanazawa Medical University Hospital
Kanazawa University Hospital
Kansai Denryoku Hospital
Kansai Medical University Hospital
Kansai Medical University Medical Center
Kansai Rosai Hospital
Kashiwa Kousei General Hospital
Kasugai Municipal Hospital
Kawakita General Hospital
Kawasaki Medical School Hospital
Kawasaki Medical School Kawasaki Hospital
Kawasaki Municipal Hospital
Kawasaki Municipal Ida Hospital
Kawasaki Saiwai Hospital
Keio University Hospital
Keiyukai Sapporo Hospital
Kindai University Hospital
Kindai University Nara Hospital
Kinki Central Hospital
Kiryu Kousei General Hospital
Kishiwada City Hospital
Kitaakita Municipal Hospital
Kitaharima Medical Center
Kitakyushu Municipal Medical Center
Kitano Hospital
Kitasato University Hospital
Kobe City Medical Center General Hospital
Kobe University Hospital
Kochi Health Science Center
Kochi University Hospital
Kokura Memorial Hospital
Kosei Hospital
Kouseiren Takaoka Hospital
Kumagai General Hospital
Kumamoto University Hospital
Kumamoto Regional Medical Center
Kurashiki Central Hospital
Kurume University Hospital
Kyonan Medical Center Fujikawa Hospital
Kyorin University Hospital
Kyoto University Hospital
Kyoto-Katsura Hospital
Kyushu Central Hospital
Kyushu University Hospital
Machida Municipal Hospital
Matsudo City General Hospital
Matsushita Memorial Hospital
Matsuyama Red Cross Hospital
Mie University Hospital
Minamiosaka Hospital
Minoh City Hospital
Mito Red Cross Hospital
Mitsui Memorial Hospital
Miyazaki University Hospital
Moriguchi Keijinkai Hospital
Nagahama City Hospital
Nagahama Red Cross Hospital
Nagano Municipal Hospital
Nagaoka Chuo General Hospital
Nagasaki University Hospital
Nagoya City University Hospital
Nagoya City West Medical Center
Nagoya Tokushukai General Hospital
Nagoya University Hospital
Nanpuh Hospital
Nara City Hospital
Nara Medical University Hospital
Nasu Red Cross Hospital
National Cancer Center Hospital
National Cancer Center Hospital East
National Center for Global Health and Medicine
National Defence Medical College Hospital
Nerima Hikarigaoka Hospital
New Tokyo Hospital
NHO Beppu Medical Center
NHO Chiba Medical Center
NHO Iwakuni Clinical Center
NHO Kure Medical Center
NHO Kyoto Medical Center
NHO Kyushu Cancer Center
NHO Kyushu Medical Center
NHO Matsumoto Medical Center
NHO Mito Medical Center
NHO Miyakonojo Medical Center
NHO Nagasaki Medical Center
NHO Nagoya Medical Center
NHO Okayama Medical Center
NHO Osaka Medical Center
NHO Saga Hospital
NHO Saitama Hospital
NHO Sendai Medical Center
NHO Shikoku Cancer Center
NHO Takasaki General Medical Center
NHO Tokyo Medical Center
NHO Yokohama Medical Center
Nihonkai General Hospital
Niigata Cancer Center Hospital
Niigata City General Hospital
Niigata Prefectural Shibata Hospital
Niigata University Medical & Dental Hospital
Nikko Memorial Hospital
Nippon Medical School Chiba Hokusou Hospital
Nippon Medical School Hospital
Nippon Medical School Musashi Kosugi Hospital
Nippon Medical School Tama Nagayama Hospital
Nishi Kobe Medical Center
Northern Okinawa Medical Center
NTT Medical Center Tokyo
Numazu City Hospital
Obihiro Kousei Hospital
Ogaki Municipal Hospital
Ogikubo Hospital
Ogori Daiichi General Hospital
Ohta Hospital
Ohta Nishinouchi Hospital
Oita Prefectural Hospital
Oita Red Cross Hospital
Oita University Hospital
Okayama City Hospital
Okayama Red Cross General Hospital
Okayama Saiseikai General Hospital
Okayama University Hospital
Okitama Public General Hospital
Onomichi Municipal Hospital
Osaka City General Hospital
Osaka City University Hospital
Osaka General Medical Center
Osaka International Cancer Institute
Osaka Medical College Hospital
Osaka Police Hospital
Osaka Red Cross Hospital
Osaka University Hospital
Osaki City Hospital
Otemae Hospital
Otsu City Hospital
Rinku General Medical Center
Saga Prefectural Hospital Koseikan
Saga University Hospital
Saiseikai Fukuoka General Hospital
Saiseikai Karatsu Hospital
Saiseikai Kyoto Hospital
Saiseikai Noe Hospital
Saiseikai Utsunomiya Hospital
Saiseikai Yamaguchi General Hospital
Saiseikai Yokohama Tobu Hospital
Saitama Medical University International Medical Center
Saitama Medical University Saitama Medical Center
Sakai City Medical Center
Saku Central Hospital
Sapporo Medical University Hospital
Seikei-kai Chiba Medical Center
Seirei Hamamatsu General Hospital
Sendai City Hospital
Sendai Kosei Hospital
Shiga General Hospital
Shiga University of Medical Science Hospital
Shimane University Hospital
Shin Takeo Hospital
Shinko Hospital
Shinshu University Hospital
Shizuoka Cancer Center
Shizuoka City Shizuoka Hospital
Shizuoka General Hospital
Showa University Hospital
Southern Tohoku General Hospital
St. Luke's International Hospital
St. Marianna University School of Medicine Hospital
St. Mary's Hospital
Steel Memorial Yawata Hospital
Suita Municipal Hospital
Tachikawa Hospital
Tagawa Municipal Hospital
Takatsuki Red Cross Hospital
Teikyo University Chiba Medical Center
Teikyo University Hospital
Teikyo University Hospital Mizonokuchi
Teine Keijinkai Hospital
Tenri Hospital
The Hospital of Hyogo College of Medicine
The Jikei University Daisan Hospital
The Jikei University Hospital
Tochigi Cancer Center
Toda Central General Hospital
Toho University Ohashi Medical Center
Toho University Omori Medical Center
Toho University Sakura Medical Center
Tohoku University Hospital
Tokai University Hachioji Hospital
Tokai University Hospital
Tokai University Tokyo Hospital
Tokushima Red Cross Hospital
Tokushima University Hospital
Tokyo Dental College Ichikawa General Hospital
Tokyo Medical and Dental University Hospital
Tokyo Medical University Hachioji Medical Center
Tokyo Medical University Hospital
Tokyo Medical University Ibaraki Medical Center
Tokyo Metropolitan Cancer and Infectious Diseases Center Komagome Hospital
Tokyo Metropolitan Tama Medical Center
Tokyo University Hospital
Tokyo Women's Medical University Hospital
Tokyo Women's Medical University Medical Center East
Tokyo Women's Medical University Yachiyo Medical Center
Tonan Hospital
Toshima Hospital
Tottori Prefectural Central Hospital
Tottori University Hospital
Toyama Prefectural Central Hospital
Toyama University Hospital
Toyonaka Municipal Hospital
Toyota Kosei Hospital
Toyota Memorial Hospital
Tsuchiura Kyodo Hospital
Tsukuba University Hospital
Tsuruoka Municipal Shonal Hospital
University Hospital, Kyoto Prefectural University of Medicine
University of the Ryukyus Hospital
Wakayama Medical University Hospital
Yamagata Prefectural Central Hospital
Yamagata University Hospital
Yamaguchi University Hospital
Yamanashi Prefectural Central Hospital
Yamanashi University Hospital
Yao Municipal Hospital
Yokkaichi Hospital
Yokohama City Municipal Hospital
Yokohama City University Hospital
Yokohama City University Medical Center
Yonezawa City Hospital
Yuai Memorial Hospital

(Total 344 institutions)

## Patient background

Tables 1, 2, 3, 4, 5, 6, 7, 8.

## I. Results of endoscopically treated patients in 2014

Tables 9, 10, 11, and Figs. 1, 2, 3.

## II. Results in patients treated with chemotherapy and/or radiotherapy in 2014

Tables 12, 13 and Figs. 4, 5, 6.

## III. Results in patients who underwent esophagectomy in 2014

Tables 14, 15, 16, 17, 18, 19, 20, 21, 22, 23, 24, 25, 26, 27, and Figs. 7, 8, 9, 10, 11, 12, 13, 14, 15

**Table 27:** Cause of death

Cause of death	Cases (%)
Death due to recurrence	1806 (62.0)
Death due to other cancer	231 (7.9)
Death due to other disease (with recurrence)	65 (2.2)
Death due to other disease (without recurrence)	402 (13.8)
Death due to other disease (recurrence unknown)	12 (0.4)
Operative death*	39 (1.3)
Postoperative hospital death**	65 (2.2)
Unknown	291 (10.0)
Total of death cases	2911

Operative mortality rate: 0.75%

*Operative death means death within 30 days after operation in or out of hospital

**Hospital death is defined as death during the same hospitalization, regardless of department at time of death. Hospital mortality rate: 2.0%

**Table:** 

Follow-up period (months)	
Median (min.–max.)	55.29 (0.07–78.78)
